# Bone mineral density and bone remodeling markers in chronic low back pain patients with active discopathy: A case-control exploratory study

**DOI:** 10.1371/journal.pone.0196536

**Published:** 2018-06-29

**Authors:** Stéphanie Teboul-Coré, Christian Roux, Didier Borderie, Sami Kolta, Marie-Martine Lefèvre-Colau, Serge Poiraudeau, François Rannou, Christelle Nguyen

**Affiliations:** 1 Université Paris Descartes, Sorbonne Paris Cité, Paris, France; 2 Service de Rééducation et de Réadaptation de l’Appareil Locomoteur et des Pathologies du Rachis, Hôpitaux Universitaires Paris Centre-Groupe Hospitalier Cochin, Assistance Publique-Hôpitaux de Paris, Paris, France; 3 Service de Rhumatologie B, Hôpitaux Universitaires Paris Centre-Groupe Hospitalier Cochin, Assistance Publique-Hôpitaux de Paris, Paris, France; 4 INSERM UMR 1153, Centre de Recherche Épidémiologie et Statistique Paris Sorbonne Cité (CRESS), ECaMO Team, Paris, France; 5 Service de Biochimie, Hôpitaux Universitaires Paris Centre-Groupe Hospitalier Cochin, Assistance Publique-Hôpitaux de Paris, Paris, France; 6 INSERM UMR 1124, Laboratoire de Pharmacologie, Toxicologie et Signalisation Cellulaire, Faculté des Sciences Fondamentales et Biomédicales, UFR Biomédicale des Saints-Pères, Paris, France; 7 Institut Fédératif de Recherche sur le Handicap, Paris, France; Medical College of Wisconsin, UNITED STATES

## Abstract

**Objective:**

We aimed to compare bone mineral density (BMD) and bone remodeling markers in chronic low back pain (cLBP) patients with and without active discopathy (Modic 1 changes).

**Design:**

We conducted a single center case-control exploratory study. For 18 months, all patients referred to a tertiary care physical medicine and rehabilitation department in France were consecutively screened. Patients fulfilling the inclusion criteria were prospectively enrolled. Cases were defined as cLBP patients with lumbar active discopathy detected on MRI and controls as cLBP patients without active discopathy. Bone mineral density (BMD) at the spine, femoral neck and total femur was assessed by dual-energy X-ray absorptiometry, and bone remodeling markers were assessed in fasting serum samples. Overall, 37 cLBP patients (13 cases and 24 controls) fulfilled inclusion criteria and were included.

**Results:**

The median age was 42.0 years (Q1-Q3: 36.0–51.0) and mean (SD) LBP duration 72.3 (57.4) months. We found that BMD and levels of bone remodeling markers in cLBP patients did not differ with and without active discopathy.

**Conclusion:**

Our results do not support the association between active discopathy and systemic bone fragility.

## Introduction

Chronic low-back pain (cLBP) is a burden on patients and health and social care systems [1], [2]. Identifying the source of cLBP is challenging because of the poor correlation with anatomopathological findings and powerful interactions with psychosocial factors [3]. In the past 3 decades, the use of MRI has contributed to identifying structural changes of vertebral-endplate subchondral bone adjacent to intervertebral degenerative disc disease, more specifically associated with cLBP than other structural changes [4] and classified as Modic changes (MC) [5]–[7]. Type 0 MC in vertebral-endplate subchondral bone corresponds to normal bone, type 1 MC to edema, type 2 MC to fatty degeneration [8], and type 3 MC to sclerosis [9].

Type 1 MC has been shown to be more closely associated with cLBP than the other types [10] and with a specific clinical and biological cLBP phenotype: cLBP patients with type 1 MC usually present acute flare of previously unremarkable cLBP, more often have pain late at night or in the morning and longer morning stiffness, and show no evidence of spondyloarthropathy [11]; also, high-sensitivity C-reactive protein level is increased [12]. These features support a concept of “active discopathy” [13], which shares similarities with osteoarthritic flares in the knee joint.

The etiopathogenic mechanisms of the condition remain controversial, and hypotheses include increased biomechanical or biochemical stresses (8) occurring with a predisposing genetic background (9). Intervertebral disc “activation” could reflect vertebral-endplate subchondral bone microtrauma related to the loss of the intervertebral disc ability to absorb repeated shocks (5). However, type 1 MC has also been observed in the absence of advanced intervertebral disc degenerative disease [14]–[16], so in some patients, vertebral-endplate subchondral bone may primarily promote “activation” of the intervertebral disc [17]. Consistently, histopathological analysis of type 1 MC vertebral-endplate subchondral bone has demonstrated active bone lesions including disruptions and fissures, trabeculous bone thickening and increased number of osteoblasts and osteoclasts [5]. Histomorphometric analyses of bone biopsies support high bone turnover in type 1 MC. The expression of some factors promoting osteoclast activation, such as osteoclast-associated receptor, is also increased in intervertebral disc [18], and men with low bone mineral density (BMD) have shown a high prevalence of intervertebral degenerative disc disease [19].

We hypothesized that changes involving adjacent vertebral-endplate subchondral bone in patients with cLBP with active discopathy could be due to systemic bone fragility. We designed a case-control exploratory study to compare BMD and levels of bone remodeling markers in cLBP patients with and without active discopathy.

## Patients and methods

### Study design

This single-center case–control exploratory study was performed in a tertiary care center (Rehabilitation Department, Rheumatology Institute, Cochin Hospital, Paris, France).

### Patient selection

All patients referred to the department were consecutively screened from December 2014 to May 2016. Patients fulfilling the following inclusion criteria and who agreed to participate were prospectively enrolled: male sex, age ≥ 18 and < 65 years, Caucasian, cLBP defined as a daily LBP persisting for ≥ 3 months, lumbar MRI ≤ 6 months, lumbar type 1 MC at a single level with preserved intervertebral disc height (intervertebral space narrowing < 50%, maximal height of the intervertebral disc visually estimated on mid-sagittal T2-weighted MRI and described as a percentage of the nearest normal above disc height) for cases, and lumbar type 0 MC for controls. Exclusion criteria were female sex, history of back surgery, cLBP related to spondyloarthropathy, infection, fracture or tumor, Scheuermann disease according to Sorensen criteria [20], spinal deformity including scoliosis and kyphosis, spondylolisthesis ≥ grade 2, types 2 and 3 MC, type 1 MC at L5-S1 level, body mass index ≥ 30 kg/m^2^, alcoholic consumption ≥ 3 units/day, current or past history of cigarette smoking ≥ 30 pack-year, current oral treatment with glucocorticoids ≥ 7.5 mg/day for ≥ 3 months, current anti-osteoporotic treatment and current or past history of metabolic, chronic kidney, liver or malabsorption diseases inducing osteoporosis.

### Imaging

Lumbar MRI with T1-, T2- and short tau inversion recovery (STIR)-weighted sequences ≤ 6 months were available for all patients. All lumbar MRI images were assessed by one investigator (STC) for presence and level of types 0, 1, 2 and 3 MC. Frontal and sagittal views of the spine in standing patients were recorded with the EOS system (EOS imaging, Paris, France), and the presence of vertebral fractures was assessed.

### BMD assessment

BMD was assessed by dual-energy X-ray absorptiometry by using a Hologic QDR 4500 A device (Hologic Inc. Waltham) at the lumbar spine (from L2 to L4) and the left hip (total femur and femoral neck) in antero-posterior projection. The device was calibrated by measuring a spine phantom each day when a patient underwent scanning and at least 3 times a week throughout the study. All exams were performed in a standardized manner, according to the manufacturer’s recommendations, by the same operator (SK) who had more than 10 years’ experience performing dual-energy X-ray absorptiometry. BMD of upper (without type 1 MC) and lower (with and without type 1 MC for cases and controls, respectively) L4 vertebra was evaluated. The whole vertebral height was divided into fixed-height spacing lines and the total number of lines was divided by 2. Reproducibility of the L4 hemivertebra BMD measure was assessed in 10 patients with measurements taken 3 times by the same observer (SK). The mean coefficient of variation of L4 hemivertebra BMD measure was 0.43% and the intraclass correlation coefficient (ICC) was 0.997 (95% confidence interval 0.991 to 0.999). The results are given as mean (SD) BMD (g/cm^2^). Mean (SD) time elapsed between lumbar MRI and BMD assessment was 93.4 (68.6) days with type 0 MC and 121.1 (71.6) days with type 1 MC (Table 1).

**Table 1:** Patients’ demographics and cLBP characteristics at inclusion.

Patients and cLBP characteristics	Modic 0 n = 24	Modic 1 n = 13	All patients n = 37
**Patients demographics**			
Age (years), median (Q1-Q3)	42.0 (37.5–47.3)	41.0 (33.0–62.0)	42.0 (36.0–51.0)
BMI (kg/m2), mean (SD)	23.2 (2.4)	26.3 (3.2)	24.3 (3.1)
Full or part-time employed, n (%)	18/24 (75.0)	10/13 (76.9)	28/37 (75.7)
**cLBP characteristics**			
Duration of cLBP (months), mean (SD)	63.7 (51.1)	88.2 (66.7)	72.3 (57.3)
Lumbar pain in the past month (VAS, 0–100 mm), mean (SD)	47.5 (25.2)	47.6 (20.9)	47.5 (23.5)
Radicular pain in the past month (VAS, 0–100 mm), mean (SD)	30.6 (33.1)	8.1 (18.4)	22.7 (30.1)
Handicap in the past month (VAS, 0–100 mm), mean (SD)	47.3 (24.9)	48.1 (20.8)	47.6 (23.252)
Quebec score (0–100), mean (SD)	33.3 (14.9)	37.5 (13.3)	34.7 (14.3)
Reproduction of pain during Valsalva maneuvers, n (%)	8/24 (33.3)	8/13 (61.5)	16/37 (43.2)
Morning stiffness ≥ 30 minutes, n (%)	3/24 (12.5)	5/13 (38.5)	8/37 (21.6)
Morning stiffness (minutes), mean (SD)	11.0 (17.2)	28.5 (33.9)	17.1 (25.4)
Worst painful moments at night or in the morning, n (%)	7/24 (29.2)	6/13 (46.2)	13/37 (35.1)
Improvement with NSAIDs, n (%)	8/22 (36.4)	8/12 (66.7)	16/34 (47.1)

BMI: body mass index, cLBP: chronic low back pain, n: absolute frequency, NSAIDs: non-steroidal anti-inflammatory drugs, SD: standard deviation and VAS: visual analog scale.

### Peripheral blood samples

Fasting serum samples were collected at baseline (between 7 and 9 am, before breakfast and at least 48 hours after non-steroidal anti-inflammatory drug intake) and stored at -40°C. We determined serum concentrations of bone remodeling markers: serum C-terminal cross-linked type I telopeptide for bone resorption and procollagen type I N-terminal propeptide, osteocalcin and bone-specific alkaline phosphatase for bone formation.

### Ethics approval

The study was carried out in accordance with L.1123-6 article of the French Health Code. The study protocol was approved by the local ethics committee (Comité consultatif de Protection des Personnes en Recherche Biomédicale de l’Île-de-France 1). Informed consent was obtained from all subjects.

### Statistical analyses

Data analyses involved the use of MYSTAT v12.02.00. For descriptive analyses, qualitative variables are reported with absolute and relative frequencies, and quantitative variables with mean (SD) or median (quartile 1–3 [Q1–Q3]). Normal distribution of quantitative data was assessed by the Shapiro-Wilk test. Groups were compared by 2-sample *t* test for normally distributed data and Mann Whitney test for non-normally distributed data. To compare qualitative data, we used chi-square test. The ICC was calculated by using MedCalc v16.8.4. Bonferroni correction was used for multiple comparisons (21 comparisons). Statistical significance was set at p<0.0024.

## Results

### Patient recruitment

**Fig 1:**
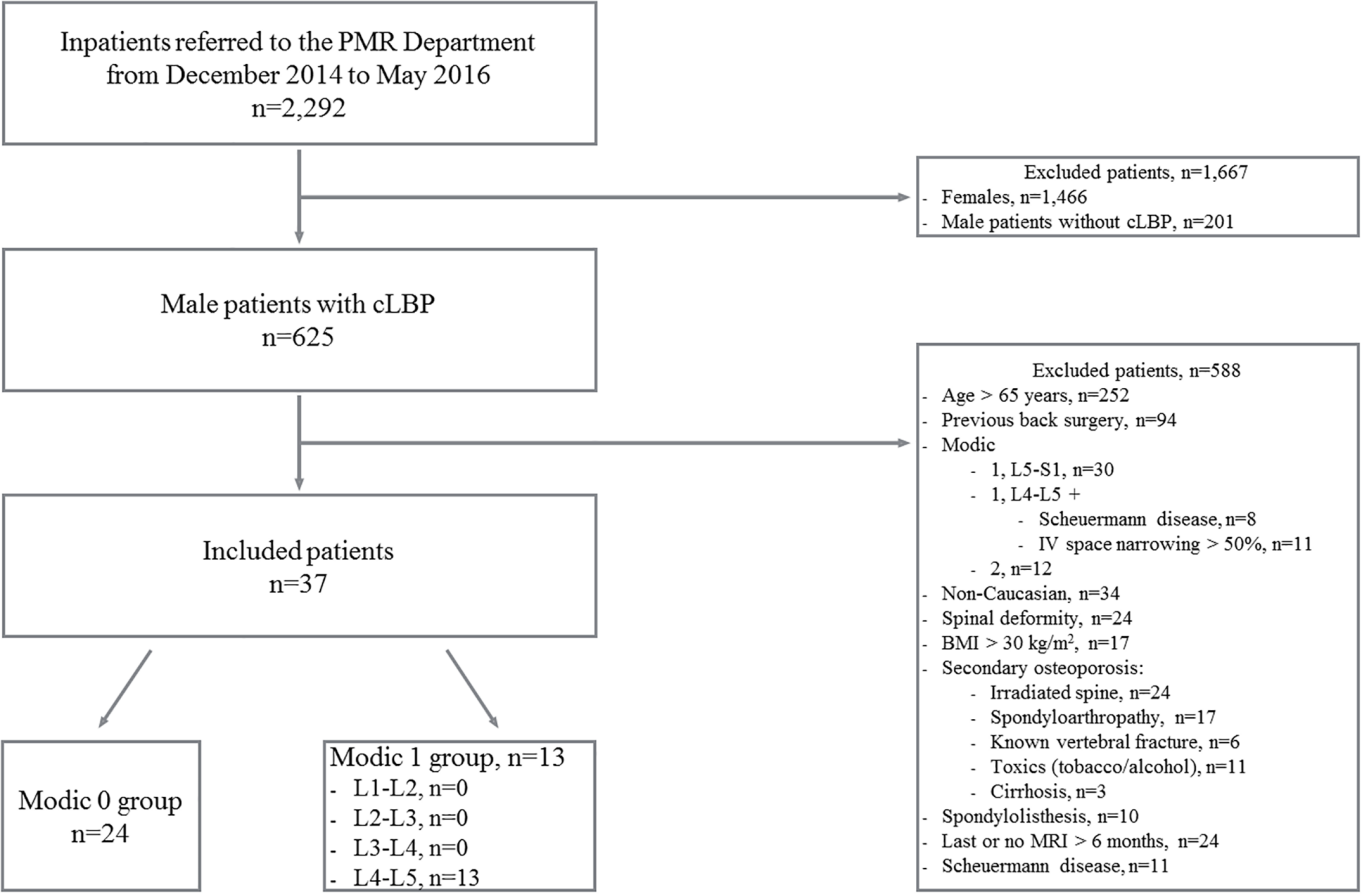
Flow chart of patients with chronic low back pain (cLBP) screened from December 2014 to May 2016 and reasons for non-inclusion. BMI: body mass index.

From December 2014 to May 2016, 2,292 inpatients were prospectively screened. In total, 1,466 patients were excluded because they were women and 201 because they were in hospital for reasons other than cLBP. Data for 525 males with cLBP were analyzed; 37 met the inclusion criteria and were prospectively enrolled: 24 without and 13 with active discopathy (Fig 1).

### Patient characteristics

At the time of inclusion, the median age was 42.0 years (Q1-Q3: 36.0–51.0) Tables 1, 2, 3, 4 and 5. Overall, 28/37 (75.7%) patients were employed. The mean (SD) LBP duration was 72.3 (57.3) months, and mean (SD) LBP intensity (VAS, 0–100 mm) in the past month was 47.5 (23.5) mm. The mean (SD) Quebec score was 34.7 (14.3). Reproduction of pain during Valsalva maneuvers was more frequent with type 1 than 0 MC (8/13 [61.5%] *vs* 8/24 [33.3%], respectively), morning stiffness duration was longer (28.5 [33.9] *vs* 11.0 [17.2] min, respectively), and the worse painful moment at night or in the morning was more frequent (6/13 [46.2%] vs 7/24 [29.2%]).

**Table 2:** Patients’ treatments at inclusion.

Treatments	Modic 0 n = 24	Modic 1 n = 13	All patients n = 37
**Current treatments, n (%)**			
Analgesics	16/24 (66.7)	5/13 (38.5)	21/37 (56.8)
Non-opioids	16/24 (66.7)	5/13 (38.5)	21/37 (56.8)
Weak opioids	9/24 (37.5)	4/13 (30.8)	13/37 (35.1)
Strong opioids	3/24 (12.5)	0/13 (0)	3/37 (8.1)
NSAIDs	4/24 (16.7)	3/13 (23.1)	7/37 (18.9)
Vitamin D	1/24 (4.2)	0/13 (0)	1/37 (2.7)
Lumbar bracing	5/24 (20.8)	4/13 (30.8)	9/37 (24.3)
**Previous treatment with intradiscal glucocorticoid injection, n (%)**	0/24 (0)	2/13 (15.4)	2/37 (5.4)

n: absolute frequency.

‘Weak opioids’ include codeine, dihydrocodeine and tramadol.

‘Strong opioids’ include morphine, diamorphine, fentanyl, buprenorphine, oxymorphone, oxycodone and hydromorphone.

**Table 3:** Patients’ physical examination findings at inclusion.

Physical examination findings	Modic 0 n = 24	Modic 1 n = 13	All patients n = 37
Modified Schöber test (cm), mean (SD)	15+5.5 (4.1)	15+5.2 (1.8)	15+5.4 (3.4)
Fingertip-to-floor test (cm), mean (SD)	24.1 (15.3)	19.0 (15.2)	22.3 (15.3)
Pain at disc pressure, n (%)	2/24 (8.3)	1/13 (7.7)	3/37 (8.1)
Increased lumbar pain in hyperextension, n (%)	10/24 (41.7)	8/13 (61.5)	18/37 (48.6)
Lasègue’s sign, n (%)	4/24 (16.7)	1/13 (7.7)	5/37 (13.5)

n: absolute frequency and SD: standard deviation.

**Table 4:** Patients’ biological findings at inclusion.

Biological findings, mean (SD)	Modic 0 n = 24	Modic 1 n = 13	All patients n = 37
HsCRP (mg/L)	2.3 (4.3)	1.2 (1.7)	1.9 (3.6)
Creatinine (μmol/L)	81.0 (11.5)	82.5 (11.0)	81.6 (11.2)
ALP (U/L)	62.9 (16.8)^a^	54.8 (11.6)	59.9 (15.5)
TSH (mUI/L)	1.8 (1.2)^a^	1.6 (0.8)	1.7 (1.0)
Free testosterone (pmol/L)	28.9 (9.5)^a^	26.7 (10.6)	28.1 (9.8)
Total testosterone (nmol/L)	19.1 (5.0)^b^	18.1 (5.8)	18.8 (5.2)

ALP: alkaline phosphatase, HsCRP: high sensitivity C-reactive protein and SD: standard deviation.

^a^n = 23

^b^n = 22

**Table 5:** Time elapsed between MRI and BMD assessment, at inclusion.

	Modic 0 n = 24	Modic 1 n = 13	All patients n = 37
**Time elapsed between MRI and BMD assessment, (days) mean (SD**)	93.4 (68.6)^b^	121.1 (71.6)	103.7 (70.0)

SD: standard deviation.

### MRI and EOS findings

Type 1 MC involved the L4-L5 level for all cases (Fig 1). No group showed prevalent vertebral fracture on EOS imaging Table 6.

**Table 6:** Comparisons of demographical risk factors for osteoporosis, prevalent vertebral fractures and bone mineral density between Modic 0 and Modic 1 cLBP patients between Modic 0 and Modic 1 cLBP patients.

	Modic 0 n = 24	Modic 1 n = 13	All patients n = 37	p-value
**Demographical risk factors**				
Previous fracture, n (%)	4/24 (29.2)	2/13 (15.4)	9/37 (24.3)	1.000
Previous low trauma fracture, n (%)	0/24 (0)	0/13 (0)	0/37 (0)	1.000
Family history of hip fracture, n (%)	1/24 (4.2)	0/13 (0)	1/37 (2.7)	1.000
Current smoking (pack-year), mean (SD)	7.5 (9.7)	6.6 (8.7)	7.2 (9.2)	0,785
**Vertebral fracture, n (%)**	0/24 (0)	0/13 (0)	0/37 (0)	1.000
**Bone mineral density (g/cm**^**2**^**), mean (SD)**				
Lumbar spine (from L2 to L4)	1.029 (0.135)	1.061 (0.100)	1.041 (0.123)	0.463
L2	1.045 (0.131)	1.033 (0.108)	1.041 (0.122)	0.776
L3	1.035 (0.151)	1.034 (0.126)	1.034 (0.141)	0.981
L4	1.012 (0.135)	1.108 (0.096)	1.046 (0.130)	0.029
Upper part of L4	0.946 (0.158)	1.019 (0.138)	0.971 (0.153)	0.170
Lower part of L4	1.085 (0.134)	1.187 (0.111)	1.121 (0.134)	0.042
Total femur	0.989 (0.130)	1.021 (0.158)	1.000 (0.139)	0.506
Femoral neck	0.833 (0.122)	0.839 (0.119)	0.835 (0.119)	0.88
**T-scores (SD), mean (SD)**				
Lumbar spine (from L2 to L4)	-0.8 (1.3)	-0.4 (0.9)	-0.6 (1.2)	-
Total femur	-0.5 (1.1)	-0.4 (1.2)	-0.5 (1.1)	-
Femoral neck	-1.2 (1.1)	-1.3 (1.1)	-1.3 (1.1)	-

cLBP: chronic low back pain, n: absolute frequency and SD: standard deviation.

### BMD

The groups did not differ in BMD assessed at the lumbar spine, total femur and femoral neck. The mean (SD) BMD for L4 total vertebra and its lower part was higher with type 1 than 0 MC but not significantly: 1.108 (0.096) versus 1.012 (0.135) g/cm^2^, p = 0.029, and 1.187 (0.111) versus 1.085 (0.134) g/cm^2^, p = 0.042, respectively. Prevalent risk factors for osteoporosis (current smoking, alcohol excess and parental history of hip fracture) did not differ between the 2 groups Table 6.

### Biological parameters

Mean concentrations of serum parameters that affect bone metabolism and bone remodeling markers did not differ between the 2 groups Table 7. With type 0 and 1 MC, serum levels of 25-hydroxy-vitamin D3 were low: 16.1 (9.3) versus 17.0 (6.5) ng/mL, p = 0.763.

**Table 7:** Comparisons of bone remodeling markers between Modic 0 and Modic 1 cLBP patients.

Biological parameters	Modic 0 n = 24	Modic 1 n = 13	All patients n = 37	p-value
**Biological parameters that influence bone metabolism, mean (SD)**				
Abumine-corrected calcium (mmol/L)	2.4 (0.1)	2.4 (0.1)	2.4 (0.1)	0.115
Phosphorus (mmoL/L)	1.0 (0.2)	1.0 (0.2)	1.0 (0.2)	0.542
25-hydroxy-vitamin D3 (ng/mL)	16.1 (9.3)^a^	17.0 (6.5)	16.4 (8.3)	0.763
Parathyroid hormone (pmol/L)	2.9 (1.1)^a^	2.4 (1.0)	2.7 (1.1)	0.065
**Bone formation markers, mean (SD)**				
Procollagen type I N-terminal propeptide (ng/mL)	30.1 (4.1)	33.2 (3.6)	33.6 (4)	0.115
Osteocalcin (ng/mL)	22.2 (4.8)	19.7 (4.2)	21.3 (4.7)	0.119
Bone-specific alkaline phosphatase (U/L)	24.3 (7.5)^a^	21.1 (4.9)	23.1 (6.8)	0.178
**Bone resorption markers, mean (SD)**				
Serum C-terminal cross-linked telopeptide of type I collagen (pg/mL)	511.2 (165.8)	441.0 (199.6)	486.6 (178.8)	0.260

SD: standard deviation.

^a^n = 23

## Discussion

In our case-control exploratory study of 37 cLBP patients, BMD and levels of bone remodeling markers did not differ between patients with and without active discopathy. Our results suggest no specific systemic osseous phenotype associated with active discopathy.

Our inclusion criteria were a major strength of our study; previous studies addressing the same topic did not exclude patients with coincidental bone fragility. Because BMD and bone remodeling markers are highly variable in middle-age women [21]–[23], we included only men. Similarly, we exclude patients over 65 years old because of the increased risk of osteoporosis with aging [24]. Moreover, intervertebral degenerative disc disease features such as osteophytes [25], [26] and vertebral endplate subchondral bone sclerosis [27] are common in this population and can artefactually increase BMD at the lumbar spine. We excluded non-Caucasian and obese patients because BMD reference curves are inaccurate and poorly determined in these populations [28]. We also excluded patients with a history of back surgery, Scheuermann disease and spinal deformity for whom measures of BMD are difficult to interpret. Finally, we excluded patients with type 1 MC at the L5-S1 level because of the technical difficulty in minimizing the low-back lordosis (19,20).

We found no significant difference between type 1 and 0 MC groups in total lumbar spine, total femur and femoral neck BMD. Only one case-control study has addressed the question of BMD in cLBP patients with MC [29] and found no significant difference between cases (n = 11) and controls (n = 10) in total lumbar spine BMD. In 7 cases of MC identified at the L3-L4 level, mean BMD was higher at the affected level than the adjacent, unaffected cephalad level [29]. Unlike our study, in the previous study, all types of MC were included and no femoral BMD was assessed, which limits the interpretation of systemic bone status. Surprisingly, we observed a decrease in BMD from L2 to L4 in controls, which may be related to the sampling.

The relationship between cLBP and BMD has not been well documented in the literature. Only one study reported the mean BMD of each lumbar vertebra in cLBP women and did not highlight an inverted lumbar BMD gradient [30]. Clinical trials have suggested the efficiency of zoledronic acid [31] and pamidronate [32], [33], long-acting bisphosphonates, in reducing the intensity of cLBP in the short term among patients with MC. From our results, the efficiency of bisphosphonates may relate more to their anti-inflammatory effects [34] than to their inhibitory effect on bone resorption [35].

We had concerns that measurement of whole vertebral body BMD by dual-energy X-ray absorptiometry may mask local changes in vertebral BMD [36]. To improve the sensitivity of measures for local changes in BMD, we measured BMD in the upper part (without type 1 MC) and lower part (with type 1 MC) of L4. BMD of L4 whole vertebra and its lower part was higher with type 1 than 0 MC. Although not significant, this observation is somewhat unexpected. In other inflammatory conditions affecting the spine, such as spondyloarthropathies, bone-marrow oedema is associated with low BMD [37]. Our finding of high lumbar BMD at the level of active discopathy may reflect the co-existence of inflammatory and sclerotic changes [27]. Using EOS imaging, we identified osteosclerotic changes of the lower part of L4 in 7/13 patients with type 1 MC, which reflects mixed type 1 to 3 MC. We believe these vertebral-endplate subchondral bone changes result more from intervertebral disc degeneration than systemic bone disease.

This hypothesis is further supported by the concept of “internal disc disruption” [38]. Repeated trauma to the intervertebral disc leads to the production of proinflammatory mediators in the *nucleus pulposus*. The breach of the endplate by the nucleus pulposus as a foreign body may induce an autoimmune reaction and cause MC [39]. Furthermore, intervertebral disc disease, by promoting unusual biomechanical conditions on vertebral-endplate subchondral bone, could lead to local bone changes. However, the cross-sectional design of our study did not allow for determining the successive stages of disc disease “activation,” and whether vertebral-endplate subchondral bone edema could promote further disc degeneration [40].

Mean concentrations of serum parameters that affect bone metabolism and bone remodeling markers did not differ between type 1 and 0 MC groups. Blood samples were obtained under strict conditions to limit misinterpretation of levels of bone turnover markers, which show intra-individual variation throughout the day and under different physiological circumstances [41]. Both bone formation and resorption markers were assessed under the hypothesis of high bone turnover similar to that observed in inflammatory diseases [42]. Only a few studies searched for a specific osseous biological phenotype associated with active discopathy. Low levels of vitamin D cause increased parathyroid hormone level leading to increased bone turnover, which increases the risk of micro-fractures in the endplate. A Danish study investigated vitamin D levels in 40 patients with MC. Unexpectedly, MC were more frequent in patients with normal vitamin D levels than in those with vitamin D levels less than 25 nmol/L [43]. In the present study, vitamin D levels were low with both type 1 and 0 MC, unlike in the Danish study, showing a mean (SD) vitamin D level of 23.6 (10.4) ng/mL in 152 cLBP patients, whatever the MC type. A national French study reported a mean (SD) vitamin D level of 23.6 (10.0) ng/mL in a healthy population in Paris [44]. Low vitamin D level could play a role in reported symptoms in cLBP contributing to diffuse pain and bone muscle and weakness [45].

Our study has several limitations. Our findings pertain to only a specific subset of cLBP patients: we took several steps to limit biases that could arise, which resulted in a high number of exclusion criteria and therefore a proportional restriction of the study population. Another limitation was multiple testing, which implied a risk of coincidental findings. We limited this risk by using Bonferroni correction. In addition, our sample size was small and was not defined by power calculation but rather by the pragmatic and exploratory design of our study. The lack of power could explain in part the lack of association between active discopathy-associated cLBP and an osseous phenotype. Finally, the cross-sectional design of our study did not allow for assessing a potential causative association between BMD and bone remodeling marker levels and changes affecting vertebral-endplate subchondral bone. Using dual-energy X-ray absorptiometry, we were not able to assess qualitative micro-architectural vertebral-endplate subchondral bone changes but rather only quantitative changes.

In conclusion, BMD and bone remodeling marker levels in cLBP patients do not differ between those with and without active discopathy. The results from our exploratory study do not support an association of active discopathy and systemic bone fragility. However, the small sample size was a limitation to the interpretation of the data. Further studies should investigate qualitative changes affecting vertebral-endplate subchondral bone adjacent to active discopathy.

## References

[1] GBD 2015 Disease and Injury Incidence and Prevalence Collaborators. Global, regional, and national incidence, prevalence, and years lived with disability for 310 diseases and injuries, 1990–2015: a systematic analysis for the Global Burden of Disease Study 2015. Lancet Lond Engl. 2016 10 8;388(10053):1545–602. 10.1016/S0140-6736(16)31678-6 PMC5055577 27733282

[2] Palazzo C, Ravaud J-F, Papelard A, Ravaud P, Poiraudeau S. The burden of musculoskeletal conditions. PloS One. 2014;9(3):e90633 doi: 10.1371/journal.pone.0090633 24595187 10.1371/journal.pone.0090633 PMC3942474

[3] Andersson GB. Epidemiological features of chronic low-back pain. Lancet Lond Engl. 1999 8 14;354(9178):581–5. 10.1016/S0140-6736(99)01312-4 10470716

[4] Jensen TS, Karppinen J, Sorensen JS, Niinimäki J, Leboeuf-Yde C. Vertebral endplate signal changes (Modic change): a systematic literature review of prevalence and association with non-specific low back pain. Eur Spine J. 2008;17(11):1407–22. doi: 10.1007/s00586-008-0770-2 18787845 10.1007/s00586-008-0770-2 PMC2583186

[5] De Roos A, Kressel H, Spritzer C, Dalinka M. MR imaging of marrow changes adjacent to end plates in degenerative lumbar disk disease. Am J Roentgenol. 1987;149(3):531–4. 3497539 10.2214/ajr.149.3.531

[6] Modic MT, Masaryk TJ, Ross JS, Carter JR. Imaging of degenerative disk disease. Radiology. 1988;168(1):177–86. doi: 10.1148/radiology.168.1.3289089 3289089 10.1148/radiology.168.1.3289089

[7] Nguyen C, Poiraudeau S, Rannou F. Vertebral subchondral bone. Osteoporos Int. 2012;23(8):857–60. 10.1007/s00198-012-2164-x 23179569

[8] Modic MT, Steinberg PM, Ross JS, Masaryk TJ, Carter JR. Degenerative disk disease: assessment of changes in vertebral body marrow with MR imaging. Radiology. 1988;166(1):193–9. 3336678 10.1148/radiology.166.1.3336678

[9] Perilli E, Parkinson IH, Truong L-H, Chong KC, Fazzalari NL, Osti OL. Modic (endplate) changes in the lumbar spine: bone micro-architecture and remodelling. Eur Spine J. 2014;1–9. 10.1007/s00586-014-3455-z 25063369

[10] Jensen MC, Brant-Zawadzki MN, Obuchowski N, Modic MT, Malkasian D, Ross JS. Magnetic resonance imaging of the lumbar spine in people without back pain. N Engl J Med. 1994;331(2):69–73. doi: 10.1056/NEJM199407143310201 8208267 10.1056/NEJM199407143310201

[11] Nguyen C, Bendeddouche I, Sanchez K, Jousse M, Papelard A, Feydy A, et al Assessment of ankylosing spondylitis criteria in patients with chronic low back pain and vertebral endplate Modic I signal changes. J Rheumatol. 2010;37(11):2334–9. doi: 10.3899/jrheum.100165 20716662 10.3899/jrheum.100165

[12] Rannou F, Ouanes W, Boutron I, Lovisi B, Fayad F, Macé Y, et al High-sensitivity C-reactive protein in chronic low back pain with vertebral end-plate modic signal changes. Arthritis Care Res. 2007;57(7):1311–5. 10.1002/art.22985 17907216

[13] Nguyen C, Poiraudeau S, Rannou F. From Modic 1 vertebral-endplate subchondral bone signal changes detected by MRI to the concept of “active discopathy”. Ann Rheum Dis. 2015 8;74(8):1488–94. doi: 10.1136/annrheumdis-2015-207317 25977562 10.1136/annrheumdis-2015-207317

[14] Luoma K, Vehmas T, Grönblad M, Kerttula L, Kääpä E. Relationship of Modic type 1 change with disc degeneration: a prospective MRI study. Skeletal Radiol. 2009 3;38(3):237–44. doi: 10.1007/s00256-008-0611-8 19096840 10.1007/s00256-008-0611-8

[15] Ma Z, Ding W, Shen Y, Sun Y, Yang D, Xu J. [The study on the relationship between modic change and disc height together with lumbar hyperosteogeny]. Zhonghua Wai Ke Za Zhi. 2013 7;51(7):610–4. 24256586

[16] Kanna RM, Shetty AP, Rajasekaran S. Patterns of lumbar disc degeneration are different in degenerative disc disease and disc prolapse magnetic resonance imaging analysis of 224 patients. Spine J Off J North Am Spine Soc. 2014 2 1;14(2):300–7. 10.1016/j.spinee.2013.10.042 24231779

[17] Revel M, Poiraudeau S, Lefèvre-Colau M-M, Mayoux-Benhamou M-A. La discopathie destructrice rapide. Rev Rhum. 2000;67:266–9.

[18] Torkki M, Majuri M-L, Wolff H, Koskelainen T, Haapea M, Niinimäki J, et al Osteoclast activators are elevated in intervertebral disks with Modic changes among patients operated for herniated nucleus pulposus. Eur Spine J. 2016;25(1):207–16. doi: 10.1007/s00586-015-3897-y 25813008 10.1007/s00586-015-3897-y

[19] Fabreguet I, Fechtenbaum J, Briot K, Paternotte S, Roux C. Lumbar disc degeneration in osteoporotic men: prevalence and assessment of the relation with presence of vertebral fracture. J Rheumatol. 2013;40(7):1183–90. doi: 10.3899/jrheum.120769 23729808 10.3899/jrheum.120769

[20] Sørensen KH. Scheuermann’s juvenile kyphosis: clinical appearances, radiography, aetiology, and prognosis. Munksgaard; 1964.

[21] Slemenda CW, Hui SL, Longcope C, Wellman H, Johnston CC. Predictors of bone mass in perimenopausal women: a prospective study of clinical data using photon absorptiometry. Ann Intern Med. 1990;112(2):96–101. 2294827 10.7326/0003-4819-112-2-96

[22] Nilas L, Christiansen C. Bone mass and its relationship to age and the menopause. J Clin Endocrinol Metab. 1987;65(4):697–702. doi: 10.1210/jcem-65-4-697 3654915 10.1210/jcem-65-4-697

[23] Watts NB. Clinical utility of biochemical markers of bone remodeling. Clin Chem. 1999;45(8):1359–68. 10430819

[24] Schuit SCE, van der Klift M, Weel AE a. M, de Laet CEDH, Burger H, Seeman E, et al Fracture incidence and association with bone mineral density in elderly men and women: the Rotterdam Study. Bone. 2004 1;34(1):195–202. 14751578 10.1016/j.bone.2003.10.001

[25] Oishi Y, Shimizu K, Katoh T, Nakao H, Yamaura M, Furuko T, et al Lack of association between lumbar disc degeneration and osteophyte formation in elderly Japanese women with back pain. Bone. 2003;32(4):405–11. 12689684 10.1016/s8756-3282(03)00031-0

[26] Ito M, Hayashi K, Yamada M, Uetani M, Nakamura T. Relationship of osteophytes to bone mineral density and spinal fracture in men. Radiology. 1993;189(2):497–502. doi: 10.1148/radiology.189.2.8210380 8210380 10.1148/radiology.189.2.8210380

[27] Muraki S, Yamamoto S, Ishibashi H, Horiuchi T, Hosoi T, Orimo H, et al Impact of degenerative spinal diseases on bone mineral density of the lumbar spine in elderly women. Osteoporos Int. 2004;15(9):724–8. doi: 10.1007/s00198-004-1600-y 14997287 10.1007/s00198-004-1600-y

[28] Wahner HW, Fogelman I. The evaluation of osteoporosis: dual energy X-ray absorptiometry in clinical practice. Br J Radiol. 1994;67(801):921–921.

[29] Briggs AM, O’Sullivan PB, Foulner D, Wark JD. Vertebral bone mineral measures and psychological wellbeing among individuals with modic changes. Clin Med Insights Case Rep. 2012;5:35–41. doi: 10.4137/CCRep.S9209 22493564 10.4137/CCRep.S9209 PMC3320114

[30] Ahn S, Song R. Bone mineral density and perceived menopausal symptoms: factors influencing low back pain in postmenopausal women. J Adv Nurs. 2009 6;65(6):1228–36. doi: 10.1111/j.1365-2648.2009.04983.x 19374677 10.1111/j.1365-2648.2009.04983.x

[31] Koivisto K, Kyllönen E, Haapea M, Niinimäki J, Sundqvist K, Pehkonen T, et al Efficacy of zoledronic acid for chronic low back pain associated with Modic changes in magnetic resonance imaging. BMC Musculoskelet Disord. 2014;15(1):64. 24588905 10.1186/1471-2474-15-64 PMC3996022

[32] Poujol D, Ristori JM, Dubost JJ, Soubrier M. Efficacy of pamidronate in erosive degenerative disk disease: A pilot study. Joint Bone Spine. 2007 12;74(6):663–4. doi: 10.1016/j.jbspin.2007.04.007 17913553 10.1016/j.jbspin.2007.04.007

[33] Cecchetti S, Pereira B, Roche A, Deschaumes C, Abdi D, Coudeyre E, et al Efficacy and safety of pamidronate in Modic type 1 changes: study protocol for a prospective randomized controlled clinical trial. Trials. 2014;15(1):1. 24716739 10.1186/1745-6215-15-117 PMC3984426

[34] Pennanen N, Lapinjoki S, Urtti A, Mönkkönen J. Effect of liposomal and free bisphosphonates on the IL-1β, IL-6 and TNFα secretion from RAW 264 cells in vitro. Pharm Res. 1995;12(6):916–22. 7667201 10.1023/a:1016281608773

[35] Hughes DE, MacDonald BR, Russell RG, Gowen M. Inhibition of osteoclast-like cell formation by bisphosphonates in long-term cultures of human bone marrow. J Clin Invest. 1989;83(6):1930 doi: 10.1172/JCI114100 2524504 10.1172/JCI114100 PMC303914

[36] Briggs AM, Perilli E, Parkinson IH, Wrigley TV, Fazzalari NL, Kantor S, et al Novel assessment of subregional bone mineral density using DXA and pQCT and subregional microarchitecture using micro-CT in whole human vertebrae: applications, methods, and correspondence between technologies. J Clin Densitom. 2010;13(2):161–74. doi: 10.1016/j.jocd.2010.01.120 20347368 10.1016/j.jocd.2010.01.120

[37] Briot K, Durnez A, Paternotte S, Miceli-Richard C, Dougados M, Roux C. Bone oedema on MRI is highly associated with low bone mineral density in patients with early inflammatory back pain: results from the DESIR cohort. Ann Rheum Dis. 2013;72(12):1914–9. doi: 10.1136/annrheumdis-2012-201845 23161904 10.1136/annrheumdis-2012-201845

[38] Crock HV. Internal disc disruption. A challenge to disc prolapse fifty years on. Spine. 1986 8;11(6):650–3. 3787337

[39] Ma X-L, Ma J-X, Wang T, Tian P, Han C. Possible role of autoimmune reaction in Modic Type I changes. Med Hypotheses. 2011;76(5):692–4. doi: 10.1016/j.mehy.2011.01.035 21339054 10.1016/j.mehy.2011.01.035

[40] Kerttula L, Luoma K, Vehmas T, Grönblad M, Kääpä E. Modic type I change may predict rapid progressive, deforming disc degeneration: a prospective 1-year follow-up study. Eur Spine J. 2012;21(6):1135–42. doi: 10.1007/s00586-012-2147-9 22249308 10.1007/s00586-012-2147-9 PMC3366121

[41] Wheater G, Elshahaly M, Tuck SP, Datta HK, van Laar JM. The clinical utility of bone marker measurements in osteoporosis. J Transl Med. 2013;11(1):1. 23984630 10.1186/1479-5876-11-201 PMC3765909

[42] Baum R, Gravallese EM. Bone as a target organ in rheumatic disease: impact on osteoclasts and osteoblasts. Clin Rev Allergy Immunol. 2015;1–15. 10.1007/s12016-015-8515-6 PMC4809775 26411424

[43] Johansen JV, Manniche C, Kjaer P. Vitamin D levels appear to be normal in Danish patients attending secondary care for low back pain and a weak positive correlation between serum level Vitamin D and Modic changes was demonstrated: a cross-sectional cohort study of consecutive patients with non-specific low back pain. BMC Musculoskelet Disord. 2013;14(1):1. 23497097 10.1186/1471-2474-14-78 PMC3608086

[44] Chapuy M-C, Preziosi P, Maamer M, Arnaud S, Galan P, Hercberg S, et al Prevalence of vitamin D insufficiency in an adult normal population. Osteoporos Int. 1997;7(5):439–43. 9425501 10.1007/s001980050030

[45] Straube S, Moore AR, Derry S, McQuay HJ. Vitamin D and chronic pain. Pain. 2009;141(1–2):10–3. 19084336 10.1016/j.pain.2008.11.010

